# Early parietofrontal network upregulation relates to future persistent deficits after severe stroke—a prospective cohort study

**DOI:** 10.1093/braincomms/fcab097

**Published:** 2021-05-04

**Authors:** Winifried Backhaus, Hanna Braaß, Focko L Higgen, Christian Gerloff, Robert Schulz

**Affiliations:** Department of Neurology, University Medical Center Hamburg-Eppendorf, 20246 Hamburg, Germany

**Keywords:** resting-state, functional connectivity, intraparietal sulcus, fMRI, coupling

## Abstract

Recent brain imaging has evidenced that parietofrontal networks show alterations after stroke which also relate to motor recovery processes. There is converging evidence for an upregulation of parietofrontal coupling between parietal brain regions and frontal motor cortices. The majority of studies though have included only moderately to mildly affected patients, particularly in the subacute or chronic stage. Whether these network alterations will also be present in severely affected patients and early after stroke and whether such information can improve correlative models to infer motor recovery remains unclear. In this prospective cohort study, 19 severely affected first-ever stroke patients (mean age 74 years, 12 females) were analysed which underwent resting-state functional MRI and clinical testing during the initial week after the event. Clinical evaluation of neurological and motor impairment as well as global disability was repeated after three and six months. Nineteen healthy participants of similar age and gender were also recruited. MRI data were used to calculate functional connectivity values between the ipsilesional primary motor cortex, the ventral premotor cortex, the supplementary motor area and the anterior and caudal intraparietal sulcus of the ipsilesional hemisphere. Linear regression models were estimated to compare parietofrontal functional connectivity between stroke patients and healthy controls and to relate them to motor recovery. The main finding was a significant increase in ipsilesional parietofrontal coupling between anterior intraparietal sulcus and the primary motor cortex in severely affected stroke patients (*P *<* *0.003). This upregulation significantly contributed to correlative models explaining variability in subsequent neurological and global disability as quantified by National Institute of Health Stroke Scale and modified Rankin Scale, respectively. Patients with increased parietofrontal coupling in the acute stage showed higher levels of persistent deficits in the late subacute stage of recovery (*P *<* *0.05). This study provides novel insights that parietofrontal networks of the ipsilesional hemisphere undergo neuroplastic alteration already very early after severe motor stroke. The association between early parietofrontal upregulation and future levels of persistent functional deficits and dependence from help in daily living might be useful in models to enhance clinical neurorehabilitative decision making.

## Teaser

Backhaus et al. used resting-state functional imaging of the ipsilesional parietofrontal network in severely impaired acute stroke patients and reported increased connectivity between anterior intraparietal sulcus and primary motor cortex compared to controls and an association of this connectivity with subsequent persistent disability in the late subacute stage of recovery.

## Graphical Abstract

**Graphical Abstract:**
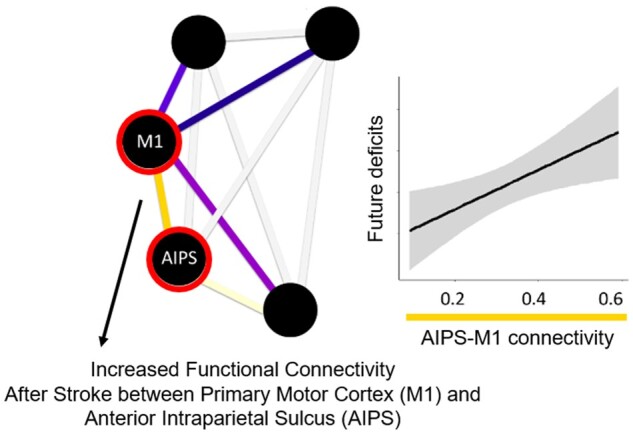

## Introduction

Neuroimaging studies have significantly enhanced our understanding of time- and recovery-dependent alterations of local brain activation and inter-regional connectivity after ischaemic stroke. Earlier work has primarily focussed on key areas of the frontal motor network, including the primary motor cortex (M1), the dorsal and ventral premotor cortices (PMC), and the supplementary motor area (SMA).^1^^,^^2^

Compared to this network, there is still limited knowledge of the extent to which posterior parietal cortices (PPC) and their interactions, particularly with frontal motor regions, might be altered after stroke and influence recovery processes. Studies in healthy participants had already evidenced that the PPC and its pathways linking different secondary motor areas, e.g. along the anterior intraparietal sulcus (AIPS) and caudal intraparietal sulcus (CIPS), with PMC and M1 influence cortical excitability of key motor areas^3^^,^^4^ and mediate skilled voluntary movements, such as reaching and grasping and using objects and tools.^5^^,^^6^ Several functional MRI and EEG analyses have aimed to explore stroke-related alterations of parietofrontal networks and their influence on residual motor functioning, recovery processes and training gains after stroke.^7–10^ For instance, in terms of resting-state functional MRI, Wang et al. observed a gradually increasing functional connectivity (FC) between the ipsilesional M1 and the contralesional superior parietal lobule.^11^ Later, Park et al. reported on the temporal patterns of parietofrontal FC alterations after stroke.^12^ A seed-based analysis from ipsilesional M1 revealed decreased FC to contralesional PPC areas at onset, but increased coupling estimates for ipsilesional PPC after one and six months.^12^ An increased parietofrontal coupling between PPC and ipsilesional M1 has also been reported in chronic stroke patients.^13^ However, significant associations with behavioural aspects were not detected. More recent functional MRI^14^ and EEG analyses^15^ on movement-related brain activation in chronic well-recovered stroke patients have similarly indicated an upregulation of parietofrontal coupling. Specifically, the data indicated that increased FC was particularly found in more impaired patients, potentially reflecting the brain’s attempt to recruit additional parietal brain regions to compensate for motor network disruption.

Importantly, the majority of studies on parietofrontal network alterations after stroke were based on moderate to mildly affected patients, particularly in the subacute or chronic recovery stage. To what extent the previous findings will hold true at all in severely affected patients and whether such changes in parietofrontal coupling might be already detectable in the first days after stroke remains unclear. Moreover, particularly concerning more recent findings of task-related connectivity analyses,^14^^,^^15^ the question arises whether parietofrontal upregulation is solely found under specific visuomotor grip task conditions in active brain states or whether it will already be present in the resting brain. Ultimately, whether early parietofrontal coupling information can improve correlative models to infer subsequent motor recovery awaits further investigation.

The present study was designed to explore stroke-related alterations of parietofrontal connectivity further. First-ever ischaemic stroke patients with severe motor deficits underwent early resting-state functional MRI to address FC of the ipsilesional parietofrontal motor network comprising M1, PMV, SMA, AIPS and CIPS^14^ and longitudinal clinical testing up to six months after stroke. Inter-regional FC was compared to healthy participants of similar age and gender and related to clinical measures of motor recovery. We hypothesized to detect significant alterations of parietofrontal network connectivity early after stroke and that the connectivity measures would relate to future deficits.

## Materials and methods

### Participants and clinical testing

In total, thirty severely affected first-ever stroke patients admitted to the University Medical Center Hamburg-Eppendorf were consecutively recruited between 10/2017 and 02/2020 and enrolled in this prospective cohort study. The study was approved by the local ethical commission (PV5442). Acute stroke patients (3–14 days after the incident), either still treated on the stroke unit or already relocated to the neurological ward, were included according to the following criteria: first-ever ischaemic stroke causing a severe motor deficit involving hand function, modified Rankin Scale (MRS) > 3 or Barthel index (BI) ≤ 30 or ‘early rehabilitation’ Barthel Index^16^ <30 and age ≥18 years. They provided informed consent themselves or via a legal guardian, following the ethical Declaration of Helsinki. Patients with pre-existing clinically silent brain lesions >1 cm³, pre-existing motor deficits, contraindications for MRI, psychiatric disease or drug abuse, or when German language skills were non-sufficient to understand the goal and implications of the study could not participate. A flow diagram of patient recruitment and study participation is given in Supplementary material. The patients underwent extensive longitudinal behavioural evaluation during the initial hospitalization (day 4–14) and the late sub-acute phase,^17^ after three and six months. Standardized tests included the National Institutes of Health Stroke Scale (NIHSS), the Fugl Meyer Assessment of the upper extremity (UEFM), the MRS and the BI. Structural and resting-state functional MRI was also performed during the initial hospitalization. All patients were matched with healthy control participants, without any neurological damage unrelated to healthy ageing, according to age and sex. All patients and controls were right-handed.

### Brain imaging—image acquisition

A 3T Skyra MRI scanner (Siemens, Erlangen, Germany) and a 32-channel head coil were used to acquire multimodal imaging data, including structural high-resolution T_1_-weighted images and functional resting-state images. For the T_1_-weighted sequence, a three-dimensional magnetization-prepared rapid gradient echo (3 D-MPRAGE) sequence was used with the following parameters: repetition time (TR) = 2500 ms, echo time (TE) = 2.12 ms, flip angle 9°, 256 coronal slices with a voxel size of 0.8 × 0.8 × 0.9 mm³, field of view (FOV) = 240 mm. The resting-state fMRI parameters for blood oxygenation level dependent (BOLD) contrasts were FOV = 260 mm, TR = 2 s, TE = 30 ms, a 72 × 72 × 32 matrix, voxel size 3 × 3 × 3 mm³, flip angle 90°, and 210 images. Before the resting-state scans, the participants were asked to focus on a black cross located behind the scanner, which could be viewed via a mirror. For the T_2_-weighted images, a fluid attenuated inversion recovery sequence was used with the following parameters: TR = 9000 ms, TE = 86 ms, TI = 2500 ms, flip angle 150°, 43 transversal slices with a voxel size of 0.7 × 0.7 × 3.0 mm³, FOV = 230mm. For the T_2_*-weighted images, the following parameters were used: TR = 4700 ms, TE = 392 ms, TI = 1800 ms, 192 sagittal slices with a voxel size of 0.8 × 0.8 × 0.9 mm³, FOV = 240 mm.

### Brain imaging—processing and analysis

Stroke lesions were masked in a semiautomatic fashion^18^ onto native T_1_-weighted images utilizing the lesion information from the T_1_-weighted, the T2star, and T2w images, if available. All resting-state fMRI- and T_1_-images with right-sided stroke lesions were flipped to the left hemisphere in a first step. This hemispheric flip was performed in the respective matched controls to account for the distribution of stroke lesions to the dominant and non-dominant hemispheres, in line with our previous studies.^19^

The default pre-processing pipeline for volume-based analysis within the CONN-toolbox v19.2 (SPM 12) was used for resting state fMRI images.^20^ The first ten volumes were discarded to account for magnetization equilibrium effects. During the initial pre-processing, all functional images were realigned (motion corrected), centred, slice time corrected, corrected for motion artefacts using the artefact detection tools (*ART*), and co-registered to their corresponding T_1_-weighted images. Images were identified as outliers if the head movement (in direction *x*, *y*, *z*) was more than five standard deviations from the mean intensity of the entire run or outside a 97th percentile threshold. All structural images were then centred and segmented into cerebrospinal fluid, grey and white matter, and spatially normalized to the Montreal Neurological Institute (MNI) template. Functional images were then normalized to MNI space using the deformation field from the corresponding structural images and spatially smoothed to allow for better registration and reduction of noise using a 6 mm full width at half maximum (FWHM) Gaussian kernel. After registration, every image was visually checked for possible registration errors due to the large stroke lesions. After pre-processing, motion parameters were derived from rigid-body realignment and their derivatives. Five potential noise components (average BOLD signal and the first four components in a principle component analysis of the covariance within the subspace orthogonal to the average BOLD signal) derived from cerebrospinal fluid and white matter using the a*CompCor* (anatomical component-based noise correction) procedure^21^ were regressed from the signal. The analyses did not include global signal regression to avoid potential false anti-correlations.^22^

The selected ipsilesional motor network,^14^^,^^15^ consisted of five central nodes. These were included as regions of interest (ROI) using spherical seeds with a radius of 5 mm, defined in MNI, for M1 (−38, −22, 54), SMA (−6, −4, 57), PMV (−54, 6, 32), AIPS (−38, −43, 52) and CIPS (−21, −64, 55). The mean BOLD signal time course, extracted from every ROI, was band-pass filtered between 0.008 Hz and 0.1 Hz to focus on slow-frequency fluctuations^23^^,^^24^ and used within an ROI-to-ROI analysis. For ROI-to-ROI analysis, the Fisher-transformed bivariate correlation coefficients (FC values) between each pair of ROI-BOLD-signals were calculated and used for further analysis.

### Statistical analysis

Statistical analyses of FC values and behavioural data were performed in R^25^ (version 3.6.2). For within-group analyses, the null-hypothesis for each connection was tested per group by applying one-sample *t*-tests against zero. For between-group analysis, linear models were estimated with FC values as the dependent variable (DV), GROUP as factor of interest and AGE as a covariate. *P*-values were corrected for multiple comparisons applying the false-discovery rate (FDR) correction.^26^ To describe time-dependent changes of clinical parameters (NIHSS, UEFM, MRS and BI), linear-mixed effects models with repeated measures were fitted with TIME as the factor of interest, *ID* as random effect. If available, the 3 months’ follow-up point was used, otherwise clinical data after 6 months were used. To relate FC values to clinical data, we constructed individual linear models with NIHSS/UEFM/MRS/BI at follow-up (T_2/3_) as the DV and the initial deficit at T_1_, FC as the predictor or interest and FU-TIME and AGE as covariates to adjust the target effects. Stepwise backward model simplification based on Akaike-Information-Criterion (AIC) was used to simplify the final correlative models. Model results are presented by predictor coefficients with their significances and overall explained variance of the final models. Statistical significance was set to a *P*-value of ≤0.05.

### Data availability

Although there are data sharing restrictions imposed by the ethical review board, data will be made available upon reasonable request, which includes submitting an analysis plan for a secondary project.

## Results

### Demographics and clinical characteristics

**Figure 1:**
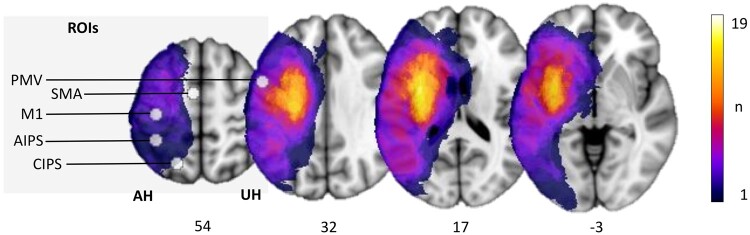
**Stroke lesions and motor network ROIs**. All masks of stroke lesions are displayed on the left hemisphere, overlaying a T_1_-weighted template in MNI space (*z*-coordinates below each slice). The colour intensity indicates the number of subjects of whom lesion voxels lay within the coloured region. Motor ROIs (M1, PMV, SMA, AIPS and CIPS) are displayed respective to the stroke lesions.

Of the initial thirty patients, eleven patients had to be excluded due to lack of functional MRI data or insufficient data quality. Nineteen patients (12 females, all right-handed, aged 75.3 ± 7.3 years, mean ± SD) were finally included in the analysis. Clinical characteristics are given in Table 1. Early clinical examination was conducted on average on day 7 (mode day 5, range 3–13) after stroke, LSA follow-up data were derived from clinical examination after 128 days on average (mode 89, range 86–217, for T_2_ and T_3_ contributing to LSA see Table 1). Linear mixed-effects models evidenced significant functional improvements over time in NIHSS (*P *<* *0.001), UEFM (*P *<* *0.001), MRS (*P *<* *0.001) and BI (*P *<* *0.001). Figure 1 gives a topographic map of the distribution of stroke lesions. This figure also illustrates the ROIs of the ipsilesional parietofrontal motor network investigated in relation to the stroke lesions.

**Table 1:** Patient characteristics at baseline and functional improvement over time

**ID**	Age	Sex	Medical History	Lesioned hemisphere/ dominance	Thombolysis/ Thrombectomy (TICI)	LVO	**Lesion volume** (ml)	Exam acute C/I	NIHSS		UEFM		MRS		BI	
									Acute	LSA	Acute	LSA	Acute	LSA	Acute	LSA
1	78	Female	HT, HC	Left/d	No/no	None	33.6	7/7	10	3	8	31	4	3	35	80
2	63	Male	HT, DM, HC	Left/d	No/no	M1	55.8	3/3	13	1	5	36	4	1	40	100
3	73	Female	HT, AF, Hthy	Left/d	Yes/yes (2B)	M1	14.4	5/5	9	3	48	65	4	3	20	100
4	73	Female	–	Right/n	Yes/yes (2A)	M1	27.6	5/5	5	2	49	62	4	1	25	80
5	79	Female	MG	Right/n	Yes/yes (2B)	M2/A1	120.4	5/6	8	2*	15	51*	5	4	10	85*
6	89	Female	HT	Right/n	Yes/no	none	2.6	4/4	7	3	5	39	5	3	40	55
7	71	Female	HT, AF	Right/n	Yes/yes (2A)	ACI/M1/A1	38.4	8/8	9	–	4	47*	5	3*	10	70*
8	76	Male	–	Right/n	Yes/yes (3)	M1/A2	101.0	5/6	11	–	4	–	5	3^†^	10	–
9	78	Male	HT, DM, AF	Right/n	Yes/yes (3)	M2	178.1	4/4	17	3	5	15	5	4	10	65
10	85	Female	HT, DM, AF, BAVR, HC	Right/n	No/yes (2B)	M1	33.5	5/5	15	14	2	4	5	5	0	0
11	78	Male	HT, AF	Left/d	No/no	M1	58.1	10/15	17	–	3	–	5	5*^,†^	10	15*^,†^
12	74	Male	HT, DM, HC, AF	Left/d	Yes/yes (2B)	M1	303.3	10/10	24	–	5	4*	5	5	5	5
13	69	Male	HT, HUR	Left/d	Yes/yes (2A)	M1	98.4	7/7	18	–	0	–	5	–	0	–
14	77	Female	HT, Asthma, AF	Right/n	Yes/no	M1	286.7	7/7	11	10*	4	4*	4	4*	20	45*
15	67	Female	OS	Right/n	Yes/no	None	7.4	8/7	11	7	6	5*	4	3	30	95
16	58	Female	HT, HC	Left/d	No/no	ACI/M1	58.4	13/13	23	–	0	–	5	–	5	–
17	80	Female	HT, AF	Left/d	No/no	M1	20.5	12/12	11	15*	–	4*	5	4*	0	30*
18	83	Female	HT, AF, CAD	Left/d	Yes/yes (3)	Carotis-T	21.6	9/9	10	–	6	–	5	–	20	–
19	80	Male	HT, DM, HC, AF, Obesity	Right/n	Yes/yes (0)	M1	108.4	7/7	16	–	6	–	5	6^†^	10	–
**Patients**	73.8 (5.8)	7 male		10 right/9 d	13 thrombolysis/ 11 thrombectomy	Modal: M1	82.5 (87.6)	Mode: 5/7	12.89 (5.17)	5.7 (5.0)	9.7 (14.5)	30.3 (23.3)	4.7 (0.5)	3.6 (1.4)	15.8 (13.0)	58.9 (34.8)
**Controls**	75.3 (7.5)	7 male		–	–	–	–		–	–	–	–	–	–	–	–

Baseline characteristics of all patients individually and averaged per group, for patients and controls. Modal values or mean values and standard deviation in brackets are given. Medical history: AF, atrial fibrillation; BAVR, biological aortic valve replacement; CAD, coronary artery disease; DM, diabetes mellitus; HC, hypercholesterinaemia; HT, hypertension; HThy, hyperthyreosis; HUR, hyperuricaemia; MG, monoclonal gammopathy; OS, osteoporosis. Hemispherical dominance: d = dominant hemisphere, n = non-dominant hemisphere was affected by the lesion, TICI: thrombolysis in cerebral infarction grading system, partial perfusion of the treated vessel is reached in grade 2B (mode). LVO, large vessel occlusion, M1/M2/A1/ACI indicate the cerebral vessels occluded. Exam time point of clinical examination (C) and imaging (I) in days after stroke. Recovery and initial scoring of four major scales: NIHSS, National Institute of Health Stroke Scale; UEFM, Upper Extremity Fugl Meyer Assessment; MRS, modified Rankin Scale; BI, Barthel-Index. Time point of data collection in the acute (day 4–14) or late sub-acute stage (LSA), either 3 or 6 months (depicted by ^*^) after stroke. †Follow-up values collected via phone.

### Parietofrontal network connectivity in the acute stage after stroke

**Figure 2:**
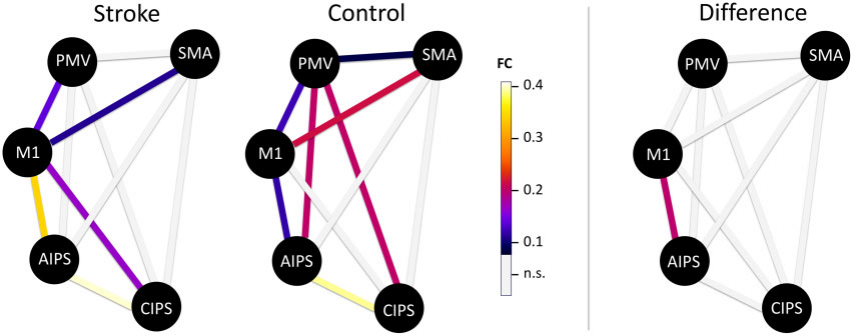
**Functional connectivity of the ipsilesional hemisphere**. Coloured lines indicate significant coupling estimates for each group (left; *P *<* *0.05, FDR-corrected for 20 tests over both groups) or significant absolute group difference for AIPS-M1 (right; *P *<* *0.05, FDR corrected for 10 tests).

Within-group analysis showed a significant coupling in the connections PMV-M1, SMA-M1, AIPS-M1 and CIPS-AIPS in stroke patients and healthy controls (Table 2, Fig. 2). Additionally, stroke patients exhibited significant coupling estimates for SMA-PMV, AIPS-PMV, and CIPS-PMV and control subjects for CIPS-M1. However, between-group analyses did not reveal a significant group difference for these connections. For ipsilesional AIPS-M1, there was a significant increase in FC in the acute stroke patients (mean 0.35) compared to healthy controls [mean 0.14, *F*(1,35) = 10.4, *P = *0.003]. This finding remained stable when excluding three patients with less severe motor deficits [UEFM ≥ 15; *F*(1,32) = 8.71, *P = *0.006], when excluding three patients with missing MRS values at follow-up [*F*(1,32) = 6.59, *P = *0.015], when excluding five patients with missing BI values at follow-up [*F*(1,30) = 7.83, *P = *0.009], when excluding one patient with a complete lesion overlap of M1 and AIPS [*F*(1,34) = 10.65, *P = *0.003, for overlap distribution with ROI masks see Supplementary Table 1] or when excluding four patients with at least partial lesion overlaps of AIPS or M1 [*F*(1,31) = 9.54, *P = *0.004].

**Table 2:** Parietofrontal network connectivity in patients and controls

	Stroke				Control				Stroke vs. control
		95% Conf.				95% Conf.			
FC	Mean	Lower	Upper	*P*-value	Mean	Lower	Upper	*P*-value	*P*-value
PMV-M1	0.16	0.08	0.24	0.001^*^	0.14	0.04	0.23	0.009^*^	0.648
SMA-M1	0.13	0.03	0.24	0.016^*^	0.21	0.13	0.29	0.001^*^	0.238
AIPS-M1	0.35	0.26	0.44	0.001^*^	0.14	0.05	0.23	0.004^*^	0.003^†^
CIPS-M1	0.17	0.07	0.28	0.003^*^	0.04	−0.06	0.14	0.430	0.053
SMA-PMV	0.05	−0.04	0.15	0.254	0.10	0.02	0.17	0.015^*^	0.459
AIPS-PMV	0.14	0.04	0.23	0.008	0.20	0.09	0.31	0.002^*^	0.389
CIPS-PMV	0.04	−0.06	0.14	0.428	0.20	0.06	0.34	0.009^*^	0.065
AIPS-SMA	0.03	−0.05	0.12	0.437	0.02	−0.06	0.10	0.588	0.840
CIPS-SMA	0.03	−0.07	0.12	0.556	0.00	−0.09	0.09	0.967	0.693
CIPS-aIPS	0.44	0.30	0.58	0.001^*^	0.41	0.31	0.50	0.001^*^	0.695

Mean values of functional connectivity (FC) are given with 95% confidence intervals for stroke patients and healthy controls. Raw *P*-values are given derived from student’s *t*-tests against 0.

* Significant values after FDR-correction for 20 tests across both groups. For group comparison, linear models were calculated across both groups and raw *P*-values of the main effect GROUP are given with † indicating significant values after FDR-correction for 10 tests.

### Influence of increased parietofrontal network connectivity on future deficits after stroke

**Figure 3:**
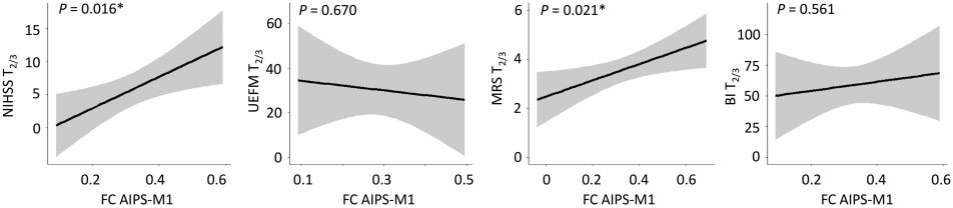
**Influence of ipsilesional AIPS-M1 functional connectivity on future persistent deficits after stroke**. Effect plots are shown for AIPS-M1 functional connectivity (FC) of the ipsilesional hemisphere contributing to the explanation of variability in follow-up NIHSS, UEFM, MRS and BI in severe stroke patients. *P*-value of FC AIPS-M1 as the predictor of interest (within-model) is given (uncorrected). There were significant associations between AIPS-M1 FC at T_1_ and MRS and NIHSS at T_2/3_ with higher FC values early after stroke found in patients which are likely to show more severe persistent deficits in follow-up, independent from the initial impairment level. A similar correlation was not detected for UEFM or BI.

Linear models were estimated to associate acute AIPS-M1 FC at T_1_ with consecutive functional scores at follow-up. We found a significant relationship between AIPS-M1 FC and global disability and neurological impairment, operationalized by means of MRS [*F*(1,13) = 6.83, *P *=* *0.021] and NIHSS at T_2/3_ [*F*(1,8) = 9.22, *P *=* *0.016, Table 3, Fig. 3], respectively. Specifically, higher FC values early after stroke were positively associated with higher MRS and NIHSS values in the late subacute stage of recovery. Importantly, these associations were independent from the initial scores for MRS and NIHSS. Herein, the addition of AIPS-M1 FC to the initial behavioural scores explained additional variance by 21% in MRS and even 48% in NIHSS at follow-up. Achieved statistical power for the detection of AIPS-M1 contributing to MRS and NIHSS was 0.60 and 0.74, respectively. For motor impairment and activity-disability, assessed by UEFM and BI, we did not detect similar FC–behaviour associations [UEFM *F*(1,9) = 0.19, *P *=* *0.670; BI *F*(1,10) = 0.36, *P *=* *0.561, Table 3, Fig. 3]. Of note, AIPS-M1 FC did not correlate alone with initial functional scores at T_1_ for NIHSS, UEFM and MRS (all *P *>* *0.34 for all three scores) but BI [*F*(1,17) = 4.74, *P *=* *0.044]. Additional exploratory regression analyses for the other parietofrontal connections revealed a similar result for CIPS-M1 at T_1_ relating to follow-up MRS [*F*(1,13) = 5.64, *P *=* *0.034] and NIHSS [*F*(1,7) = 16.21, *P *=* *0.005], but not for UEFM [*F*(1,9) = 1.45, *P *=* *0.259], or BI [*F*(1,9) = 0.12, *P *=* *0.737].

**Table 3:** Influence of AIPS-M1 FC on motor recovery after stroke

AIPS-M1				
Outcome	Predictor	Model summary		
		Coef.	*P*	*R*²
NIHSS T_2/3_	AIPS-M1	24.00	0.016^*^	0.59^*^
	NIHSS T_1_	0.45	0.208	
UEFM T_2/3_†	AIPS-M1	−21.55	0.670	0.54^*^
	UEFM T_1_	1.06	0.011^*^	
MRS T_2/3_	AIPS-M1	3.31	0.021^*^	0.59^*^
	MRS T_1_	1.48	0.009^*^	
BI T_2/3_†	AIPS-M1	37.03	0.561	0.46^*^
	BI T_1_	1.59	0.036^*^	
	Age	−2.16	0.078^†^	

Coefficients are given incl. their *P*-values (within regression model) for individual models for the four outcome variables (dependent variable) and AIPS-M1 FC at T_1_ as the main predictor of interest.

* Significant predictors or overall model fit. *R*^2^ shows multiple *R*^2^.

† For completeness, model results are given including AIPS-M1 predictor although stepwise backward model simplification resulted in a simple model with UEFM at T_1_ as the only relevant variable.

## Discussion

The main finding of the present report was a significant increase in ipsilesional parietofrontal coupling between the AIPS and M1 early after a first-ever ischaemic stroke resulting in severe motor deficits. This upregulation significantly contributed to correlative models explaining variability in subsequent neurological deficits and global disability: Patients with increased parietofrontal coupling in the acute stage showed higher levels of deficits in the late subacute stage of recovery, independent from the initial level of deficits.

Our data extend numerous previous reports showing that posterior parietal brain regions of the ipsilesional hemisphere exhibit regional activation changes and alterations of inter-regional coupling with key motor areas of the frontal lobe. Repeatedly, these key motor areas, particularly M1, SMA and PMC have been set the focus of brain activation studies. Across studies, it has been shown that increased bilateral brain activation, particularly in more severely affected patients, is a common finding after stroke. The reinstatement of lateralized and localized activation patterns has been associated with motor recovery.^2^ Brain activation in ipsilesional M1 and PMC has been positively related to residual motor functioning, both in the acute and chronic stages of recovery.^27^ To what extent posterior parietal brain regions contribute to recovery processes is still not fully understood. So far, consistent activation changes have not been detected in these locations by meta-analyses^2^ or reported by systematic reviews.^27^ One longitudinal task-related functional MRI study has reported an increase in ipsilesional superior parietal lobe brain activation in 11 moderately affected stroke patients compared to controls. Notably, more severely impaired patients were reported to show a more pronounced upregulation than mildly impaired patients.^28^ From a FC perspective, data supporting a relevant role of the PPC for motor recovery are still limited. Simulation studies have indicated that lesions along the cortical midline comprising frontal and parietal regions and the temporo-parietal junction are likely to result in largest disturbances in simulated whole-brain FC.^29^ Park et al. used resting-state functional MRI and investigated younger and less severely affected patients. The authors found a reduced parietofrontal coupling between contralesional PPC and ipsilesional M1 at onset, and an FC increase between bilateral PPC and M1 1 month after the event. After 3 months, consistent alterations were not detected anymore. After 6 months, FC between ipsilesional PPC and ipsilesional M1 coupling was enhanced again while contralesional PPC showed an FC reduction. However, an association between these alterations and behavioural aspects was not detected.^12^ Another study in chronic stroke patients reported that increased FC between ipsilesional M1 and parietal operculum predicted functional gains under a structured motor training: Patients with increased parietofrontal coupling showed less motor improvement than patients with lower coupling estimates.^10^ More recently, two task-based functional MRI^14^ and EEG^15^ studies found enhanced couplings between ipsilesional AIPS and M1 in late subacute, well-recovered stroke patients. The increase in coupling was found to be positively related to the extent of functional impairment.^15^

The present analyses now evidence a similar positive association between an enhanced ipsilesional parietofrontal connectivity and the level of subsequent persistent neurological deficits and global disability after severe ischaemic stroke. Moreover, the data show that this upregulation is already present within the first two weeks after stroke and not related to a slow and gradual evolution over time across several weeks or months. Amongst severely affected stroke patients of the present cohort, the potential of further recovery seems to be limited particularly in patients with early parietofrontal network upregulation. Or, in other words, patients with most severe deficits show strongest recruitment of parietofrontal network resources to promote recovery—an attempt which appears to be largely insufficient in the end. On a speculative note and relating to possible underlying pathophysiological mechanisms, parietal cortices might be recruited to improve M1 cortical excitability to drive the environment of excitability and neuroplasticity that accompany motor recovery after stroke. Studies in healthy participants had already shown that M1 and AIPS are functionally connected: Focal PPC stimulation was found to be capable of increasing corticospinal excitability of ipsilateral M1.^3^^,^^4^ Furthermore, diffusion-imaging-based analyses have successfully reconstructed direct cortico-cortical long-range fibres connecting AIPS with M1, herewith providing the structural bases for these concepts.^4^^,^^30^ Another possible way how the PPC might contribute to motor functioning might emerge from its contribution to corticofugal motor pathways which could potentially help to bypass the disrupted corticospinal tract (CST). Indeed, it is well known that the CST does not only originate from M1 but also from multiple secondary motor areas of the frontal and parietal lobe.^31–33^ For instance, microsimulation experiments have shown that complex movements can be elicited from M1 and the parietal lobe.^34^ It has also been reported that parietal brain regions could indirectly influence movement production through interneurons in the spinal cord.^35^ More recently, diffusion-based tractography could reconstruct corticofugal trajectories in monkeys originating from AIPS traversing the internal capsule more posteriorly than the CST from M1 or frontal premotor areas.^36^ However, the existence of such connections in humans remains relatively vague.^37^

These two concepts would consider early parietofrontal upregulation after severe stroke as an adaptive but insufficient attempt to support future motor recovery. Alternatively, an upregulation could also lead to maladaptive neuroplastic alterations after stroke and impede recovery processes. Concerning the motor domain, there is only very few data which might provide further insights: For instance, Tscherpel et al. used transcranial magnetic stimulation (TMS) to focally disturb ongoing contralesional parietal brain activity during motor tasks of varying complexity. Interestingly, they found that this inference improved motor functions already early after stroke, a finding which was stable up to 3 months. The authors argued that this finding might be mediated by an improvement of the balance of both parietal cortices helping to ameliorate neglect-related symptoms^38^ and thereby built on previous reports on neglect after stroke.^39^^,^^40^ Whether disruption of ipsilesional parietal brain activity in areas along the intraparietal sulcus would lead to similar findings or whether an upregulation by non-invasive brain stimulation^41^ would exert beneficial effects clearly remains a topic for future prospective and systematic investigations. However, based on available previous functional^9^^,^^42^ and structural data in stroke patients^30^^,^^43^ supporting the view that better parietofrontal network integrity positively associates with better behaviour after stroke, we argue that parietofrontal upregulation would constitute an adaptive helpful attempt to promote recovery: Thus, further enhancing parietofrontal connectivity—particularly in more impaired patients—might be a reasonable way to promote recovery after stroke. Interestingly, one animal study has already evidenced that sensory-parietal cortical stimulation might support recovery processes after severe capsular stroke in a rodent stroke model.^44^

There are a number of critical limitations to note. First, apart from the limited number of severely affected acute stroke patients included, we monitored oxygen saturation and heart rate during resting-state fMRI. However, we did not record any physiological measures, such as cardiac and respiratory cycle, which could potentially correct FC measures for these effects. Second, as in most stroke studies, stroke symptoms, lesion locations, and sizes were highly heterogeneous. Patients were all classified as severe stroke patients based on MRS or (early rehabilitation) BI in the acute stage. Most patients were not able to give written informed consent on their own. This was given by official legal guardians, in most cases carrying relatives. Also, the monocentric design of the study may have introduced a selection bias. In addition to severe motor deficits, attentional and executive functions were also highly restricted, possibly explaining discrepancies between our results and previous studies.^12^

Third, based on clear a-priori hypotheses,^14^^,^^15^ we focussed on a relatively small parietofrontal network in the ipsilesional hemisphere. To what extent the main finding of the increase in FC between ipsilesional AIPS and M1 is direct in nature or mediated by hidden nodes, such as contralesional cortical or subcortical brain regions, remains unclear. The consideration of an extended network including contralesional brain areas or subcortical structures such as the putamen might extend the present findings.^45^ Fourth, the group comparisons for FC data directed to achieve high specificity by means of correction for multiple testing at the cost of reduced sensitivity. Hence, the present findings for AIPS-M1 do not exclude that other connections of the parietofrontal network might undergo stroke-related changes and might also contribute to recovery processes after severe stroke. Fifth, for BI we found a significant positive association between AIPS-M1 FC and activity-related disability at T_1_. This collinearity might explain why we could not detect any relation with BI at T_2/3_. Hence, this negative finding should be interpreted with caution.

With respect to clinical application, decision making can be delicate and intricate in patients with severe stroke, especially in the early acute phase, when patients are bedridden and limited in all domains of life. The current study found an association between network properties of the parietofrontal motor network during the first days after stroke and future levels of neurological deficits and global dependence from help in daily living. Upcoming studies will have to evaluate whether this association might be found in larger clinical cohorts and whether they might be applicable in clinical decision-making.

## Supplementary material

Supplementary material is available at *Brain Communications* online.

## Funding

This research was primarily funded by the Else Kröner-Fresenius-Stiftung (2016_A214 to R.S.). Additional funding was provided by the German Research Foundation (DFG, SFB 936-C1 to C.G.) and the German Research Foundation (DFG) in cooperation with the National Science Foundation of China (NSFC) (SFB TRR-169/A3 to C.G.).

## Competing interests

The authors report no competing interests.

## Supplementary Material

fcab097_Supplementary_Data

## Abbreviations

AIPS =: anterior part of the intraparietal sulcus
CIPS =: caudal part of the intraparietal sulcus
FC =: functional connectivity
FDR =: false discovery rate
M1 =: primary motor cortex
PMV =: ventral premotor cortex
PPC =: posterior parietal cortex
SMA =: supplementary motor area
