# Supplementary material for: Early parietofrontal network upregulation relates to future persistent deficits after severe stroke—a prospective cohort study
Source: Brain Commun. 2021 May 4;3(2):fcab097. doi: 10.1093/braincomms/fcab097 (PMC8154858; doi:10.1093/braincomms/fcab097)
Supplement: fcab097_Supplementary_Data [file fcab097_supplementary_data.pdf]

Supplementary Material

Early parietofrontal network upregulation relates to future persistent deficits after severe stroke

Winifried Backhaus Ph.D. [1, †], Hanna Braaß MD [1, †], Focko L. Higgen MD [1],
Christian Gerloff MD [1] and Robert Schulz MD [1]

[1] Department of Neurology, University Medical Center Hamburg-Eppendorf, 20246 Hamburg, Germany
[†] contributed equally

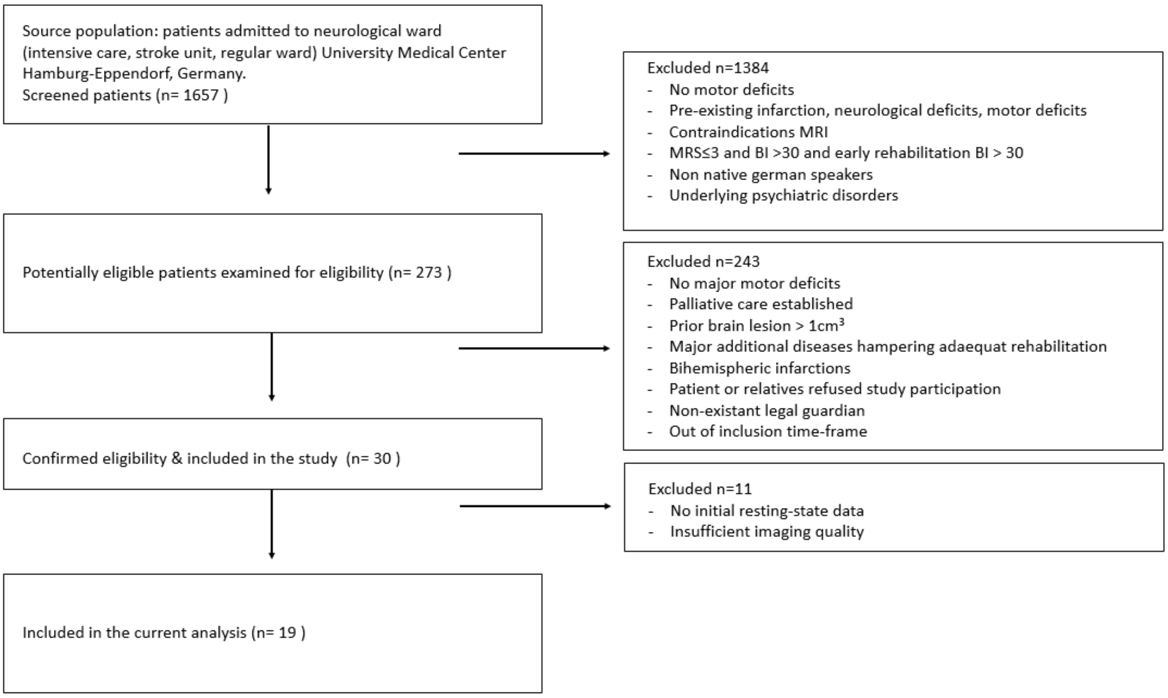

***Supplementary figure 1** | Flow diagram of patient recruitment and participation.*

Supplementary table 1 *| Overlap ROI mask and stroke lesions in % of ROI mask.*

ID	M1	PMV	SMA	AIPS	CIPS
1	0,0	0,0	0,0	0,0	0,0
2	0,0	34,6	0,0	0,0	0,0
3	0,0	0,0	0,0	0,0	0,0
4	0,0	0,0	0,0	0,0	0,0
5*	53,6	98,9	0,0	0,0	0,0
6	0,0	0,0	0,0	0,0	0,0
7	0,0	9,6	0,0	0,0	0,0
8	0,0	5,1	0,0	0,0	0,0
9*	100,0	52,8	0,0	100,0	63,4
10	0,0	0,0	0,0	0,0	0,0
11	0,0	0,0	0,0	0,0	0,0
12*	99,5	100,0	0,0	66,2	0,0
13	0,0	100,0	0,0	0,0	0,0
14	0,0	100,0	0,0	0,0	0,0
15	0,0	0,0	0,0	0,0	0,0
16	0,0	0,6	0,0	0,0	0,0
17	0,0	0,0	0,0	0,0	0,0
18	0,0	0,0	0,0	0,0	0,0
19	2,3	0,0	0,0	0,0	0,0
